# Modulation of Agrin and RhoA Pathways Ameliorates Movement Defects and Synapse Morphology in MYO9A-Depleted Zebrafish

**DOI:** 10.3390/cells8080848

**Published:** 2019-08-07

**Authors:** Emily O’Connor, George Cairns, Sally Spendiff, David Burns, Stefan Hettwer, Armin Mäder, Juliane Müller, Rita Horvath, Clarke Slater, Andreas Roos, Hanns Lochmüller

**Affiliations:** 1 John Walton Muscular Dystrophy Research Centre, MRC Centre for Neuromuscular Diseases, Institute of Genetic Medicine, Newcastle University, Newcastle upon Tyne NE1 3BZ, UK; 2 Interdisciplinary School of Health Sciences, Faculty of Health Sciences, University of Ottawa, Ottawa, ON K1H 8L1, Canada; 3 Children’s Hospital of Eastern Ontario Research Institute, University of Ottawa, Ottawa, ON K1H 8L1, Canada; 4 Institute of Cell and Molecular Biosciences, Newcastle University, Newcastle Upon Tyne NE2 4HH, UK; 5 Neurotune AG. Wagistrasse 27a, 8952 Schlieren, Switzerland; 6 Department of Clinical Neurosciences, University of Cambridge, Cambridge CB2 0QQ, UK; 7 Institute of Neuroscience, Newcastle University, Newcastle Upon Tyne NE2 4HH, UK; 8 Department of Neuropediatrics, Centre for Neuromuscular Disorders in Children, University Hospital Essen, University of Duisburg-Essen, 45122 Essen, Germany; 9 Department of Neuropediatrics and Muscle Disorders, Faculty of Medicine, Medical Center—University of Freiburg, 79106 Freiburg, Germany; 10 Centro Nacional de Análisis Genómico (CNAG-CRG), Center for Genomic Regulation, Barcelona Institute of Science and Technology (BIST), 08028 Barcelona, Spain; 11 Division of Neurology, Department of Medicine, The Ottawa Hospital, Ottawa, ON K1H 7X5, Canada

**Keywords:** myosin IXa, unconventional myosin, NT1654, fasudil, neuromuscular junction, Myo9aa, Myo9ab

## Abstract

Congenital myasthenic syndromes (CMS) are a group of rare, inherited disorders characterised by impaired function of the neuromuscular junction (NMJ). This is due to defects in one of the many proteins associated with the NMJ. In three patients with CMS, missense mutations in a gene encoding an unconventional myosin protein, MYO9A, were identified as likely causing their disorder. Preliminary studies revealed a potential involvement of the RhoA/ROCK pathway and of a key NMJ protein, agrin, in the pathophysiology of MYO9A-depletion. In this study, a CRISPR/Cas9 approach was used to generate genetic mutants of *MYO9A* zebrafish orthologues, *myo9aa/ab,* to expand and refine the morphological analysis of the NMJ. Injection of NT1654, a synthetic agrin fragment compound, improved NMJ structure and zebrafish movement in the absence of Myo9aa/ab. In addition, treatment of zebrafish with fasudil, a ROCK inhibitor, also provided improvements to the morphology of NMJs in early development, as well as rescuing movement defects, but not to the same extent as NT1654 and not at later time points. Therefore, this study highlights a role for MYO9A at the NMJ, the first unconventional myosin motor protein associated with a neuromuscular disease, and provides a potential mechanism of action of MYO9A-pathophysiology.

## 1. Introduction

The neuromuscular junction (NMJ) is a highly specialised cholinergic synapse formed between a motor axon terminal and its target skeletal muscle fibre. It acts to transmit signals from the central nervous system to the muscle to stimulate contraction for functions such as maintenance of posture, movement of limbs, and respiration. Many proteins contribute to the structure and function of the NMJ during development and throughout adult life, acting to maintain the safety factor of the NMJ, which ensures reliable and efficient signal transmission. In a rare group of disorders termed the Congenital Myasthenic Syndromes (CMS), there is genetic impairment to one of numerous genes encoding proteins that play a critical role at the NMJ [1]. In patients, this manifests as fatigable muscle weakness, along with a number of other symptoms that vary between subtypes [2].

In 2016, we identified missense mutations in *MYO9A*, a gene encoding for the unconventional myosin IXA protein [3], as the likely cause of CMS in three patients from two unrelated families. Subsequent research demonstrated an impact of MYO9A-loss on endo/exocytosis and vesicle trafficking in a nerve-cell line (NSC-34). MYO9A is a negative regulator of Ras Homolog Family Member A (RhoA) [4,5,6], and the in vitro defects described could be partly ameliorated by blocking a downstream target of the RhoA pathway, Rho-associated protein kinase (ROCK), with Y-27632. This highlighted the potential involvement of the RhoGTPase domain of MYO9A in the pathophysiology of MYO9A-CMS. As trafficking and endo/exocytosis were impaired in the absence of MYO9A, we hypothesised that this may in turn affect release of proteins at the NMJ, and thus secretomics was performed on MYO9A-depleted NSC-34 cells [5]. This revealed a significant decrease in secretion of agrin, a molecule crucial for post-synaptic NMJ formation and maintenance [5,7,8,9,10]. To further investigate the role of MYO9A, a morpholino (MO)-based model in zebrafish was generated targeting their two *MYO9A* orthologues (*myo9aa/ab*). The zebrafish knockdown embryos demonstrated impaired movement during development, and observational analysis indicated the presence of disrupted NMJ morphology. Application of an agrin fragment compound generated by Neurotune, NT1654, ameliorated the movement defects and NMJ phenotype [5,11]. While these findings provided support regarding a role for MYO9A at the NMJ and the positive potential for partial agrin-replacement by exogenous application of NT1654, the specificity of MOs and phenotypes produced have been the topic of some concern within the scientific community. This is largely due to a disparity between phenotypes observed in morphants and genetic mutants generated using techniques such as CRISPR/Cas9, as well as the frequent off-target effects induced by MO injection [12,13].

Therefore, the aims of this paper were to implement a CRISPR/Cas9-mediated approach for Myo9aa/ab knockdown in zebrafish, characterise the NMJ defects in greater detail, and to test two treatment strategies. As we hypothesised that disruption to secretion in the absence of MYO9A was related to interactions of this unconventional myosin protein with the RhoA/ROCK pathway, modulation of this pathway was also tested in the zebrafish model using the ROCK inhibitor compound fasudil. Evidence for the benefits of NT1654 application as a potential treatment and as a proof of principal for the proposed mode of action of MYO9A-loss were also obtained in greater detail, and over a longer time-period than previously performed.

## 2. Materials and Methods

### 2.1. Zebrafish Maintenance

Zebrafish (AB wild-type strain or Golden slc24a5b1/+ strain, Zebrafish International Resource Centre, Eugene, OR, USA) were maintained according to Home Office guidelines (Project License: 70/8038), with a continuous light-dark cycle (14 h light, 10 h dark). Age of zebrafish expressed as hours post fertilisation (hpf). Euthanasia of fish was performed using a 1:1 ratio of fresh system water: 4 mg/mL tricaine methanesulfonate.

### 2.2. CRISPR/Cas9-Mediated Knockdown in Zebrafish

The protocol was modified from Varshney et al. [14]. Single guide RNAs (sgRNAs) for generating CRISPR/Cas9-mediated knockdown zebrafish were designed using CRISPR scan (http://www.crisprscan.org/, date last accessed 20 February 2019 [15]). sgRNAs with the highest CRISPRscan score (efficiency score) and no predicted off-target effects were selected, with a total of 2 sgRNAs for each gene (2 × *myo9aa* exon 2, 1 × *myo9ab* exon 1, 1 × *myo9ab* exon 12). A published sgRNA against the Tyrosinase gene (*tyr)* was also included as an injected control [16]. DNA oligonucleotides that included the target region and surrounding T7 promoter sequence and tail sequence were annealed with a universal bottom strand ultramer (5′AAAAGCACCGACTCGGTGCCACTTTTTCAAGTTGATAACGGACTAGCCTTATTTTAACTTGCTATTTCTAGCTCTAAAAC-3′, Table S1). Annealing was performed using a MyTaq DNA polymerase kit as outlined in Tables S2 and S3. A Qiagen PCR purification kit was then used to purify samples according to the manufacturer’s instructions. The purified product was used as a template for a transcription reaction using a MEGAshortscript T7 kit (Invitrogen, Waltham, MA, USA) according to the manufacturer’s instructions. A total of 8 reactions for each sgRNA were pooled to increase yield prior to application to a mirVana spin column (mirVana miRNA isolation kit, ThermoFisher Scientific, Waltham, MA, USA), and subject to RNA purification according to the manufacturer’s instructions. Before injection into zebrafish embryos, the sgRNA and Cas9 reaction mix was prepared as outlined in Table S4, and heated to 37 °C for 5 min to improve knockdown efficiency as described by Burger et al. [17].

For generating ‘crispant fish’ (the F0 mosaic fish), sgRNA and Cas9 protein were injected directly into the cell at the single cell stage. Individual sgRNAs were injected to confirm ability to induce insertions/deletions, before co-injection of all 4 sgRNAs for the experimental protocol. Fish were incubated in E3 medium (5 mM NaCl, 0.17 mM KCl, 0.33 mM MgSO_4_, 0.33 mM CaCl_2_, and 0.01% methylene blue) at 28.5 °C for a maximum of 5 days, with dead fish removed daily. For experiments requiring dechorionation, pronase (*Streptomyces griseus*, Roche, Basel, Switzerland) was added to the zebrafish embryos at a final concentration of 1 mg/mL in E3 medium.

### 2.3. NT1654 Treatment

The agrin compound NT1654 was used to treat *myo9aa/ab* crispant zebrafish. This compound is a 44 kDa artificial agrin fragment developed by Neurotune AG (Switzerland, [11]). NT1654 (0.15 ng) was delivered to the embryos at the same time as sgRNA/Cas9 injection, due to the size being incompatible with water-based diffusion delivery [18], as performed previously [5]. Embryos from the same pair of fish were split into each of the three categories (wild-type, *myo9aa/ab crispant*, treated *myo9aa/ab* crispant) for each injection session to ensure fair comparisons for survival and development.

### 2.4. Fasudil Treatment

Fasudil (5 mM, Millipore Sigma, Darmstadt, Germany), a ROCK inhibitor, was used to treat the *myo9aa/ab* crispant zebrafish. The zebrafish were housed in 12-well plates, with ten or less fish per well, in E3 medium. A range of concentrations of fasudil were trialled to optimise the dose, from 1 nM to 100 µm, starting from 7 hpf and final dose-finding assessments made at 48 hpf. Based on preliminary survival rate data and assessment of chorion movements in response to each dose, a final concentration of 10 µm was used.

Fish were split into 3 treatment groups, wild-type, *myo9aa/ab* crispant, and 10 µm fasudil treated *myo9aa/ab* crispant. Treatment started at 7 hpf and was continued to 5 dpf, with solution changes daily.

### 2.5. Chorion Movements and Tactile Response Assay

Chorion movements were assessed at 24 hpf as previously described [3,5]. Briefly, embryos were recorded using a Leica stereomicroscope mounted with a Chameleon digital camera (CMLN-13s2M, FLIR Systems, Kent, UK). Recordings were made for one minute and the number of full twists performed by each fish was then manually counted from the recordings. At 48 hpf, response of zebrafish to tactile stimulation was analysed as previously described; fish classed as having a ‘severe’ phenotype were not used for movement analysis [5]. Briefly, fish were placed individually in a petri dish containing E3 medium on top of an illuminated stage, and a Canon legria hfr76 camera was clamped 7 cm above the dish. A fine pipette was used to touch the zebrafish on the back of the head and the response recorded. Room temperature remained constant at 28 °C throughout the experiment. Videos were imported into Fiji ImageJ [19] as FFmpeg movies, and then manually thresholded to allow visualisation of the zebrafish, before converting to a binary image. The Trackmate plugin [20] was then used to measure the movement of the zebrafish, with manual editing of each frame to ensure that only the zebrafish was detected and the movement identified was accurate. Values for distance travelled and average speed were exported, from which the initial acceleration could be derived. In vivo experiments were blinded prior to live recording and for image acquisition.

### 2.6. Zebrafish Whole-Mount Immunofluorescence

Fixation and staining of zebrafish was performed as described previously; fish with a severe phenotype were excluded from analysis [21]. Briefly, fish at 24, 48, and 120 hpf were dechorionated, euthanized with Tricaine, and fixed in 4% paraformaldehyde in phosphate buffered saline (PBS) overnight at 4 °C. Fish at 120 hpf were subject to Collagenase A (Millipore Sigma, 1mg/mL) treatment for 90 min prior to immunofluorescence. The presynaptic NMJ was incubated overnight at 4 °C with a mouse anti-synaptic vesicle protein 2 (SV2, 1:200, Developmental Studies Hybridoma Bank, Iowa City, IA, USA) antibody detected with secondary antibody (Alexa Fluor 594 IgG goat anti-mouse, 1:500, Life Technologies, Waltham, MA, USA). α-bungarotoxin (αBTx) conjugated to Alexa Fluor 488 was incubated with the secondary antibody for 2 h at room temperature to detect postsynaptic acetylcholine receptors (AChRs, 1:1000, ThermoFisher Scientific). Antibodies were diluted in 5% horse serum in PBS with 0.1% tween. Washes were performed using PBS with 0.1% tween. Fish were mounted in Vectashield fluorescent mounting medium (Vector Laboratories). Z-stack images encompassing the entire volume of the zebrafish tail and multiple somites around somite 15 were obtained using a 40× oil-immersion objective on a Nikon A1R confocal microscope.

### 2.7. NMJ Morphology Assessment

Fiji (ImageJ, Madison, WI, USA) was used to generate maximum intensity projections of acquired z-stack images, and measurements were obtained at the same somite level between treatment groups. At 24 hpf, myotome area was measured and the presence or absence of a central AChR cluster noted, referred to as the ‘choice point’. The distance that motor neuron axons travelled past the choice point was also manually measured, along with total and average AChR area per 100 µm^2^. At 48 and 120 hpf, myotome size was measured, along with the number of presynaptic and postsynaptic clusters per 100 µm^2^, average size of clusters and total area of clusters 100 µm^2^, as well as the number of large clusters (>20 µm^2^).

Counts and measurements of clusters were performed by automatic thresholding and conversion of images to binary before using the ‘analyse particles’ tool in Fiji. The length of the myosepta (displaying AChR-positive areas) was manually measured, along with the contact by motor neurons, giving a value for percentage of myosepta overlaid by motor neuron.

Colocalisation analysis between SV2 and αBTx-positive signals was performed on maximum intensity projections of 120 hpf zebrafish encompassing multiple somites in the field of view. The ‘EzColocalization’ Fiji plugin was used for analysis according to the protocol described by Stauffer, et al. [22]. Briefly, each fluorophore channel was subject to automatic thresholding to remove background, and the Mander’s correlation coefficient calculated to give a value between 0 and 1, reflecting the degree of co-occurrence of signals between both SV2 and αBTx, and also αBTx and SV2.

### 2.8. Statistical Analysis

Statistical analysis was performed using GraphPad Prism software (v8.0.2, BD Biosciences, San Jose, CA, USA). Data sets were first tested for normal distribution and, from these results, either nonparametric (Kruskal-Wallis with Dunn’s post hoc test) or parametric tests (one-way ANOVA with Turkey post hoc test) were applied. Outliers were identified and removed according to the ROUT method. Statistical significance was taken as *p* < 0.05. In vivo experiments were blinded prior to live recording and for image acquisition.

## 3. Results

### 3.1. Myo9aa/ab Crispant Phenotype

**Figure 1:**
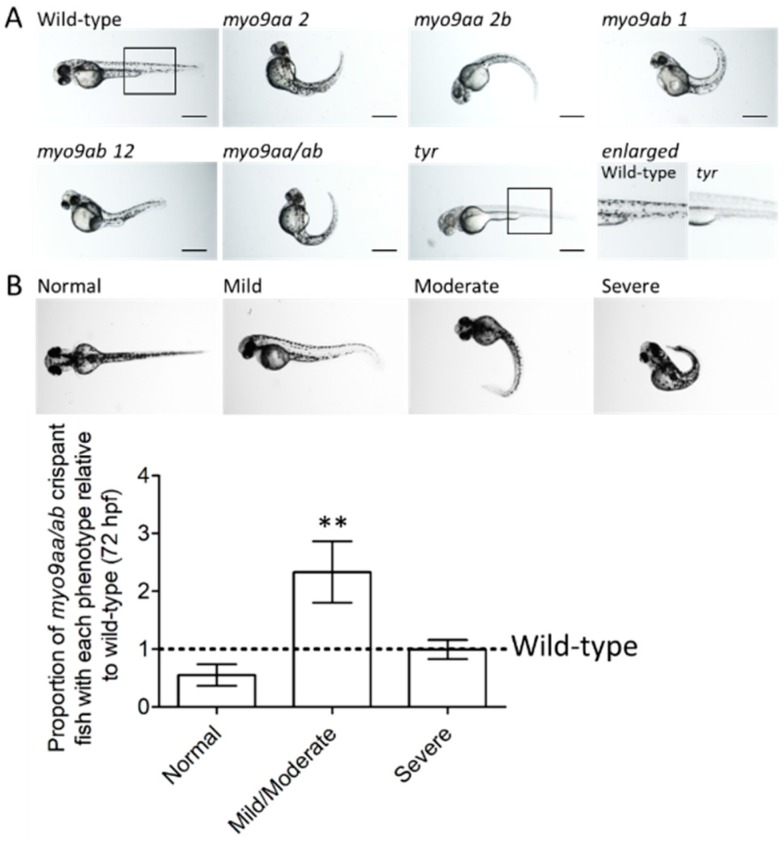
Phenotypes of *myo9aa/ab* crispant zebrafish. (**A**) Representative images of 48 hpf zebrafish injected with sgRNAs against *myo9aa* and *myo9ab* (numbers following the gene refer to exon targeted). Injection of a combination of all four sgRNAs was performed (*myo9aa/ab*), along with a control against the tyrosinase gene (*tyr*) in which no pigment can be observed, confirming knockdown success. Scale bar = 500 µm. (**B**) Images showing phenotypic classification of injected zebrafish at 72 hours post fertilisation (hpf), ranging from normal to severe. Graph shows proportion of *myo9aa/ab* crispant fish injected with all four sgRNAs (*n* = 7, including 160 fish in total) as compared to wild-types (black line, *n* = 7, including 138 fish in total). Error bars represent mean ± S.E.M, ** *p* < 0.01, paired *t*-test.

A CRISPR/Cas9 approach was used to knockdown expression of *myo9aa* and *myo9ab*. Four different sgRNAs, two against each gene (*myo9aa* and *myo9ab*), with no predicted off-target effects, were synthesised and injected into zebrafish at the one-cell stage along with the Cas9 protein. The effectiveness of each sgRNA for inducing deletions/insertions at the predicted genomic locations was confirmed using sequencing (Supplementary Figure S1). An example of the common phenotype observed at 48 hpf produced by injection of each sgRNA injected individually and in combination (termed *myo9aa/ab* crispant) is shown in Figure 1A. All injected sgRNAs induced a curved tail phenotype, as found previously in the MO-injected zebrafish [3,5]. As also observed in the morphants, there was often the presence of mild oedema, usually in the cardiac region.

Tyrosinase is a protein involved in pigment formation in fish. A previously published, sgRNA against *tyr* was included as an injected control [16]. The lack of pigment confirms the success of Cas9/sgRNA delivery and action, as highlighted in Figure 1A. Furthermore, fish injected with *tyr* sgRNA do not have a tail phenotype or presence of oedema, demonstrating that this is not a result of the CRISPR/Cas9 administration process itself.

At 72 hpf, wild-type (uninjected) and *myo9aa/ab* crispant fish were subject to analysis of gross morphology. Overall phenotype was classified as normal, mild/moderate, or severe based on degree of tail curvature, as shown in Figure 1B. Quantification of the number of *myo9aa/ab* crispant fish that reside within each group, as compared to wild-type, revealed no significant difference in proportion of normal or severe fish. There was a significant increase in the number of crispant fish with a mild/moderate phenotype, as compared to wild-type (Figure 1B).

### 3.2. Myo9aa/ab Crispant Survival and Movement in the Presence of NT1654 and Fasudil

**Figure 2:**
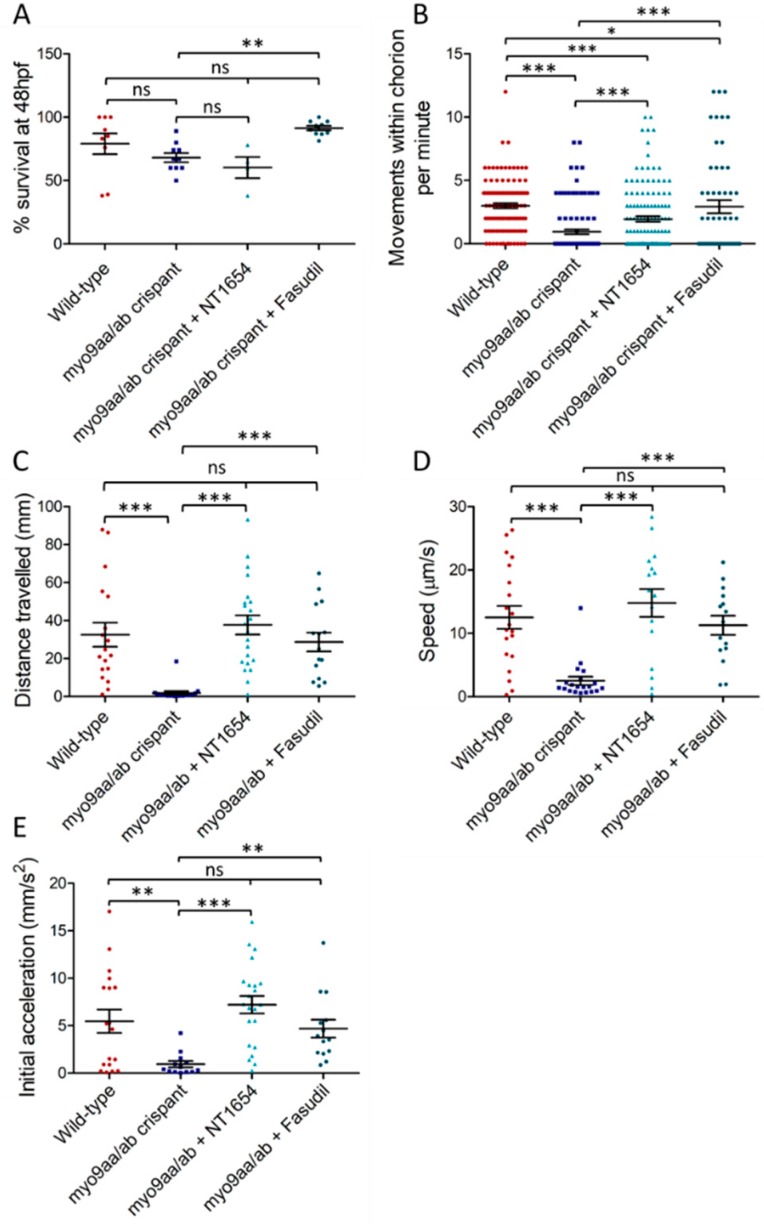
Survival and movement of *myo9aa/ab* crispant fish. (**A**) Survival of wild-type (*n* = *n* = 9 repeats), *myo9aa/ab* crispant (*n* = *n* = 10 repeats), and NT1654 (0.15 ng, *n* = 4 repeats) or fasudil-treated (10 µm, *n* = 10 repeats) crispant zebrafish at 48 hpf, expressed as a percentage of fish in each group at 0 hpf. (**B**) Full chorion movements performed by wild-type (*n* = 110 fish), *myo9aa/ab* crispant (*n* = 104 fish), and NT1654 (0.15 ng, *n* = 129 fish) or fasudil-treated (10 µm, *n* = 54 fish) crispant zebrafish at 24 hpf. (**C**) Distance, (**D**) Speed, and (**E**) Initial acceleration were quantified following a tactile-stimulation assay in the zebrafish. Wild-type (*n* = 18 fish), *myo9aa/ab* crispant (*n* = 19 fish), and NT1654 (0.15 ng, *n* = 22 fish) or fasudil-treated (10 µm, *n* = 15 fish) crispant zebrafish were subject to the assay at 48 hpf. Error bars = mean ± S.E.M. * *p* ≤ 0.05, ** *p* ≤ 0.01, *** *p* ≤ 0.001, ns = not significant, Kruskal-Wallis test.

Reduced survival is often seen when using MOs due to non-specific toxic effects [23], or due to removal of protein expression fundamental to early development. Therefore, the effect of CRISPR/Cas9-mediated Myo9aa/ab-depletion on survival was assessed (Figure 2A). At 48 hpf, *myo9aa/ab* crispant fish revealed no significant decrease in survival, as compared to wild-type fish, as opposed to results previously obtained using MO-mediated knockdown [5]. Co-injection of Cas9/sgRNA and NT1654, an agrin fragment compound, also had no significant impact on survival. Fasudil, a ROCK inhibitor drug, was added to the zebrafish medium at 7 hpf. At 48 hpf, no significant differences in survival were observed, as compared to wild-type fish; however, a significant increase in survival for *myo9aa/ab* crispants was present beyond that observed for wild-type.

Zebrafish perform well characterised movements during development at distinct time points, such as chorion coiling up to 27 hpf and the ability to respond to tactile stimulation thereafter. These movements coincide with development of the NMJ, allowing any differences that impact on NMJ signalling to be identified if they manifest as changes in behaviour.

Spontaneous movements begin in zebrafish embryos around 17 hpf, and as axon outgrowth progresses across the myotomal surface, the movements increase in frequency until they cease at 27 hpf [24,25]. The spontaneous movements are characterised by a full rotation of the embryo within the chorion, driven by the tail. Previous results from the Myo9aa/ab MO zebrafish revealed a significant reduction in chorion movements performed at 24 hpf, as compared to controls [3]. Similarly, in this study, wild-type zebrafish performed on average 2.4 rotations per minute, as compared to only 0.3 in *myo9aa/ab* crispants (Figure 2B). Treatment of crispants with NT1654 significantly increased movements at 24 hpf to 1.9 rotations per minute, although these are still significantly lower than observed for wild-type zebrafish. Fasudil also significantly improved chorion movements in Myo9aa/ab-depleted fish to 2.9 rotations per minute, although movements again remain below those found in the wild-types.

In the hours that follow the initial outgrowth of secondary motor neurons at 27 hpf, the fish gain the ability to respond to touch by swimming away from the stimulus if removed from the chorion. This swimming response increases in frequency until 36 hpf, where it peaks [25]. As zebrafish generally hatch from the chorion by 48 hpf, when the touch response should have been established in all the zebrafish, this time-point was selected for performing a touch-evoked response assay. This consisted of a touch to the back of the zebrafish head using a fine pipette tip to stimulate a rapid swim away from the stimulus, which can be recorded and analysed [5,25]. Distance travelled following the stimulus, average speed of movement, and initial acceleration (indicative of force produced by the muscle [26]) were all significantly decreased in *myo9aa/ab* crispants, as compared to wild-type fish (Figure 2C–E). Application of both NT1654 and fasudil to *myo9aa/ab* crispants rescued the movement defects, significantly improving distance, speed, and acceleration to the same level as observed in wild-type zebrafish. Overall, reduction in Myo9aa/ab using CRISPR/Cas9 produced similar effects to those observed in morphants, with a reduction in chorion movements and response to tactile stimulation, without significantly affecting survival rates. Application of an exogenous agrin fragment (NT1654) or a ROCK inhibitor (fasudil) was sufficient to provide motor improvements at both time points analysed.

### 3.3. NMJ Morphology at 24 hpf

A comprehensive morphological characterisation protocol was applied to study the NMJs of *myo9aa/ab* crispant fish at different time points during development. Time points selected encompass different stages of NMJ maturation and reflect the performance of motor tests: 24 hpf (initial outgrowth of primary motor neurons, synaptogenesis occurring, and spontaneous movement), 48 hpf (second and higher order branches of primary motor neurons present, secondary motor neuron outgrowth, response to tactile stimulation), and 120 hpf (innervation territories and coverage representative of adult patterns, larvae behaviours obtained including escape response and spontaneous swimming, maximal swimming rates achieved).

**Figure 3:**
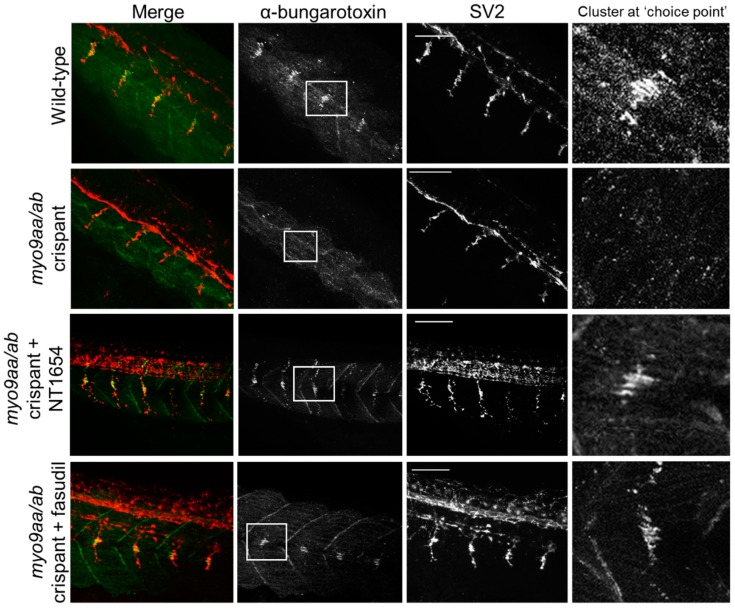
Neuromuscular junction (NMJ) morphology of *myo9aa/ab* crispant zebrafish at 24 hpf. Representative images of NMJs in wildtype, *myo9aa/ab* crispant, and NT1654 (0.15 ng) or fasudil-treated (10 µm) crispant zebrafish at 24 hpf. Acetylcholine receptors stained with αBTx (green), and motor neurons detected with an antibody against SV2 (red). White boxes demark areas enlarged in the right-hand panel, showing presence of αBTx-positive choice point clusters. Scale bar = 50 µm.

At 24 hpf, zebrafish were stained with fluorophore-conjugated αBTx to detect AChRs and an antibody against SV2 to label presynaptic motor neuron vesicles. At this stage in development, zebrafish have a cluster of AChRs at the horizontal myoseptum and this represents a pathway ‘choice point’ for extending primary motor neurons. This is clearly visible in wild-type zebrafish at this time-point, but frequently absent in the *myo9aa/ab* crispants (Figure 3, Table 1, 65% less choice point clusters than in wild-type). It should be noted that this time-point is after agrin-independent prepatterning has taken place; rather, these clusters represent receptors that have already been overlaid by motor neurons. Treatment with NT1654 increased the visibility of this cluster to 100%, as well as increased the outgrowth of motor neurons (Figure 3, Table 1). Fasudil treatment also improved the presence of the choice point cluster by 58%, but had no clear effect on motor neuron outgrowth (Figure 3, Table 1).

0000Various morphological features of NMJs in early development were quantified, as shown in Table 1. Reduction of Myo9aa/ab significantly reduced the length of neuron outgrowths past the choice point, as observed in Figure 3. It should be noted that the choice point always occurs at the horizontal myoseptum, therefore in the absence of the choice point, cluster motor neuron length was measured from the horizontal myosepta. The number of large (>20 µm^2^) AChR clusters was also significantly reduced as compared to wild-type, further reflected in the absence of the large choice point cluster in a number of the fish. There were no significant differences in the average area of the AChR clusters present, or in the total area of such clusters per 100 µm^2^; however, the size of myotomes was significantly reduced in the crispant fish. Treatment with NT1654 restored these features in the *myo9aa/ab* crispant zebrafish, including rescuing the length of neuron outgrowths past the choice point, the number of large AChR clusters and the proportion of somites with a choice point cluster. NT1654 also significantly increased the average size of AChR clusters beyond that observed in wild-type fish, and further decreased the size of myotomes. Fasudil similarly increased visibility of the choice point AChR cluster at 24 hpf, as well as improving the size of myotomes. While there was no significant improvement in the number of large AChR clusters or in the length of neuron outgrowths past the choice point, the increases in both phenotypes induced by fasudil were sufficient to restore wild-type levels. Overall, at 24 hpf, a number of pre- and postsynaptic defects to NMJ morphology can be observed in *myo9aa/ab* crispant fish, many of which were rescued by application of NT1654 or fasudil.

### 3.4. NMJ Morphology at 48 hpf

**Figure 4:**
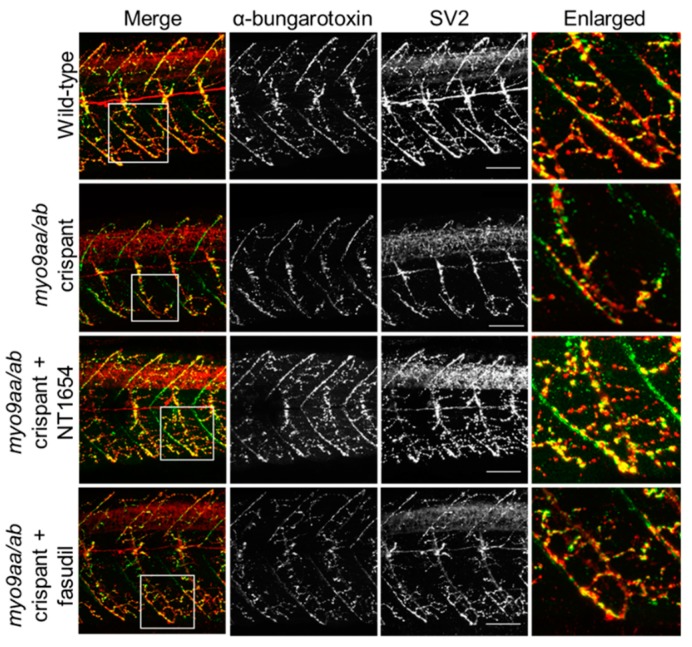
NMJ morphology of *myo9aa/ab* crispant zebrafish at 48 hpf. Representative images of NMJs in wild-type, *myo9aa/ab* crispant, and NT1654 (0.15 ng) or fasudil-treated (10 µm) crispant zebrafish at 48 hpf. Acetylcholine receptors stained with αBTx (green), and motor neurons detected with an antibody against SV2 (red). White boxes demark areas enlarged in the right-hand panel. Scale bar = 50 µm.

At the later time point of 48 hpf, motor neurons have extended down the middle of the myotome and started to contact the vertical myoseptal region. The AChR choice point cluster has dispersed and the majority of prepatterned receptors have been contacted by extending motor axons. An example of a myotome with fewer pre/postsynaptic clusters present in the crispant fish, as compared to wild-type, is shown in Figure 4. The application of NT1654 appears to provide improvement to the innervation pattern across crispant myotomes, although changes are less clear following fasudil treatment and there is an apparent lack of AChR-positive staining. These changes are quantified in Table 2.

A number of pre- and postsynaptic morphological features were also assessed at 48 hpf, as outlined in Table 2. Reflecting the impairments observed in movement at this time-point, myo9aa/ab crispant zebrafish had significantly fewer SV2-positive (presynaptic) clusters per 100 µm^2^ than wild-type fish. This was accompanied by an increase in the average cluster area; however, total presynaptic immunofluorescence per 100 µm^2^ was reduced overall. Surprisingly, despite the frequent presence of motor neurons that had not started to extend up the vertical myoseptum in crispant fish, the percentage of myosepta overlaid by motor neuron was not significantly decreased, as compared to wild-type. With regards to postsynaptic phenotypes detected with αBTx, a similar trend was observed. There was a tendency towards significantly fewer but larger clusters of AChRs, but with an overall reduction in total AChR immunofluorescence by around 50%. While myotomes in crispants were on average 700 µm^2^ smaller than those in wild-type fish, this reduction is not significant, as opposed to results from 24 hpf.

Treatment of crispants with NT1654 rescued the number of pre- and postsynaptic clusters per 100 µm^2^, as well as their average area. Improvements to the same level as wild-type were also observed for the total area of presynaptic fluorescence per 100 µm^2^; however, total αBTx area was not improved at this time-point and the number of large (>20 µm^2^) clusters was also significantly reduced, as compared to wild-type. Treatment with fasudil did not provide any additional improvements in 48 hpf zebrafish; rather, the treatment significantly reduced the number of presynaptic clusters per 100 µm^2^. Therefore, at 48 hpf, numerous defects to pre- and postsynaptic NMJ morphology were induced by depletion of myo9aa/ab, and the majority were rescued by NT1654 treatment. Fasudil application did not mediate any positive effects on NMJ morphology according to these measurements at this time-point.

### 3.5. NMJ Morphology at 120 hpf

**Figure 5:**
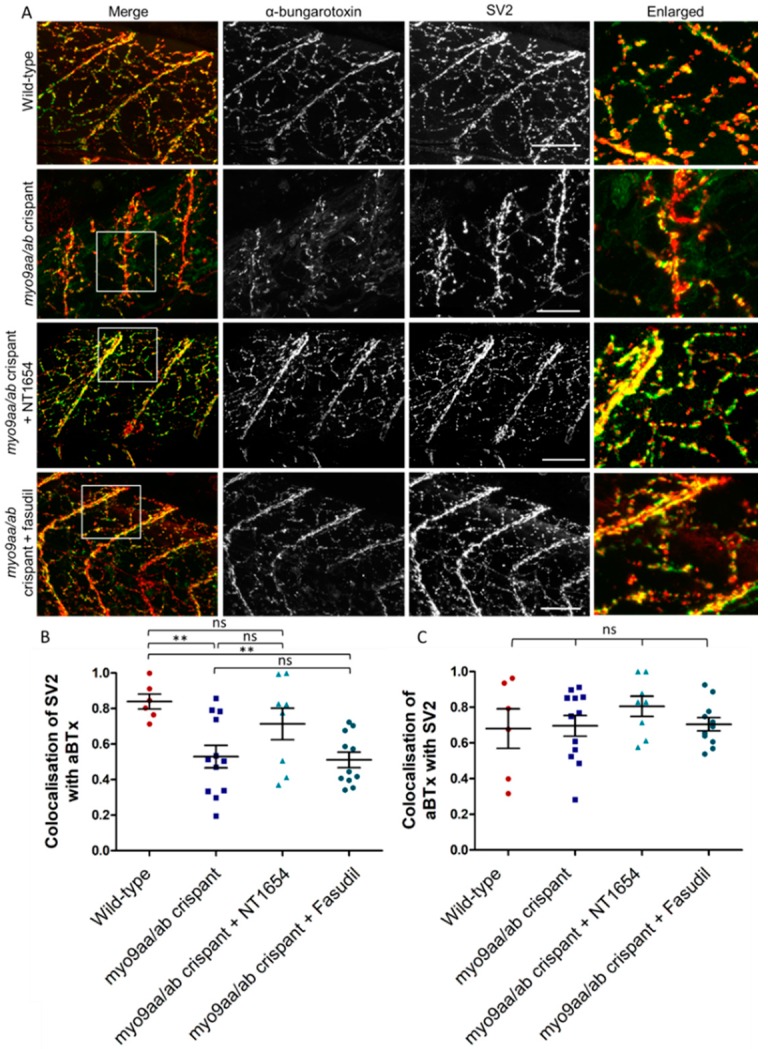
NMJ morphology of *myo9aa/ab* crispant zebrafish at 120 hpf. (**A**) Representative images of NMJs in wild-type, *myo9aa/ab* crispant, and NT1654 (0.15 ng) or fasudil-treated (10 µm) crispant zebrafish at 120 hpf. Acetylcholine receptors stained with αBTx (green), and motor neurons detected with an antibody against SV2 (red). White boxes demark areas enlarged in the right-hand panel. Scale bar = 50 µm. (**B**) Colocalisation of SV2-positive signal with αBTx and (**C**) colocalisation of αBTx with SV2-positive signal using Mander’s correlation coefficient (0 = no colocalisation, 1 = full colocalisation). Wildtype (*n* = 6 fish), *myo9aa/ab* crispant (*n* = 12 fish), crispant treated with NT1654 (0.15 ng, *n* = 8 fish), and crispant treated with fasudil (10 µm, *n* = 11 fish) were subject to analysis. Error bars = mean ± S.E.M. ** *p* ≤ 0.01 and ns = not significant, One-way ANOVA.

One of the benefits of using the CRISPR/Cas9 technique to reduce *myo9aa/ab* expression is the ability to analyse phenotypes at later time points than possible with MOs, due to the direct and permanent effects on the genomic sequence. At 120 hpf, zebrafish myotomes have a dense innervation from primary and secondary motor neurons which are in contact with AChRs. In *myo9aa/ab* crispant zebrafish, there were examples of sparsely innervated myotomes as shown in Figure 5A, with less complex branching than in controls. These features were improved by application of NT1654, but not by fasudil. Results are quantified in Table 3. Colocalisation between presynaptic and postsynaptic immunofluorescence was also quantified to determine the extent that the synaptic components overlay each other. Any myotome in which non-specific SV2 staining of the spinal cord was observed was not included in analysis, as can be observed with this antibody [27]. The amount of SV2-positive signal overlaying αBTx was significantly reduced in myo9aa/ab crispant fish, indicating that there were innervated regions lacking corresponding AChRs (Figure 5B). Application of fasudil did not improve this lack of colocalisation, with values remaining significantly below those observed in wild-type fish. On the other hand, NT1654 treatment increased the apposition of the pre- and postsynapse to a level not significantly different to wild-type. Co-occurrence of AChRs with SV2-positive signal revealed no significant differences between any treatment/genotype group, highlighting that the AChRs present were appropriately apposed with nerve terminals (Figure 5C).

NMJ morphology was also assessed at this time-point, revealing that *myo9aa/ab* crispants no longer had significantly impaired presynaptic components, as compared to wild-type. The number of postsynaptic AChR clusters per 100 µm^2^ were significantly increased, whereas average area of these clusters was significantly smaller. These alterations did not lead to a change in total AChR cluster area per 100 µm^2^. The size of myotomes was reduced in crispants, as compared to wild-type fish, as also observed at 24 hpf. Treatment with NT1654 rescued myotome size and also significantly increased the average area of SV2 clusters; however, this remained similar to the size in wild-type fish. The average area of αBTx-positive clusters decreased further than observed in untreated crispants; however, this was coupled with a significant increase in the number of αBTx clusters per 100 µm^2^. Fasudil application did not yield positive improvements in 120 hpf crispant fish, with significant disruption to all presynaptic and postsynaptic features analysed, except for the degree of myoseptal contact by motor neurons, and a non-significant increase in crispant fish myotome size.

Overall, depletion of Myo9aa/ab from zebrafish significantly affected a number of pre- and postsynaptic morphological features of developing NMJs up to 120 hpf. Many phenotypes could be rescued by NT1654-treatment and some amelioration was provided by modulation of the ROCK pathway with fasudil at earlier time-points.

## 4. Discussion

**Figure 6:**
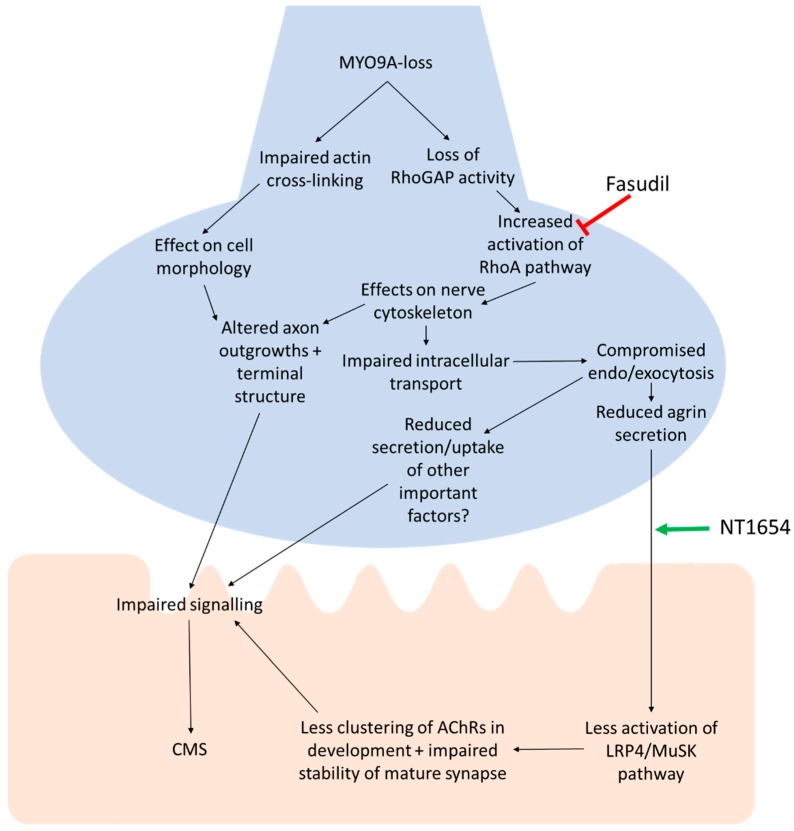
Proposed pathway of action for MYO9A-dysfunction at the NMJ. The pathway begins with loss or dysfunction of MYO9A and the predicted effect of this on a number of downstream processes, culminating in impaired neuromuscular transmission and Congenital Myasthenic Syndromes (CMS). The points of action for fasudil (ROCK inhibitor) and NT1654 (agrin-fragment) are shown. Green arrow = positive action, red arrow = inhibits action.

The NMJ is a complex synapse, with many proteins important for its development and function. The safety factor of the NMJ generally maintains its signalling ability, resulting in a consistent postsynaptic response to presynaptic release of ACh. Disruption to the morphology of the NMJ can impair this safety factor by reducing the probability of release below an effective threshold, the efficacy of postsynaptic signal amplification, the ACh clearance, or the function of AChRs, among a number of other mechanisms [28]. The role of *MYO9A*, a novel CMS-associated gene, at the NMJ is as yet unclear. The results of this study provide further evidence for the importance of this protein at the NMJ, and also support our proposed mechanism of action, as outlined in Figure 6: Loss of MYO9A reduces its action on the RhoA/ROCK pathway, downstream effects on secretion including agrin release, less clustering/stability of AChRs, resulting in impaired signal transmission and CMS. While further investigations using electrophysiology will be required to attribute physiological changes to MYO9A-loss at the NMJ, a detailed overview of NMJ morphology indicates there are at least structural changes to this cholinergic synapse that are associated with disruptions to motor behaviours in zebrafish.

Supporting our previous results obtained using a MO-mediated knockdown approach in zebrafish against the two fish orthologue, *myo9aa/ab*, crispant fish lacking expression of both of these myosin proteins displayed similar phenotypes. This included not only a curved tail, but also the presence of oedema, initially thought to result from the off-target effects produced from MO injection. MYO9A knockout mice exhibit low molecular weight proteinuria, which has recently been shown to produce oedema in early development of zebrafish—thus, this phenotype will require further investigation, as well as examination of patients for potential kidney problems [29,30].

Morphants treated with NT1654 displayed a reduced survival rate, which could have resulted in a sampling bias of surviving zebrafish for the subsequent studies. Crispant fish treated with NT1654 do not exhibit a significant reduction in survival rates, indicating the previous finding was likely due to toxic effects of co-injecting two morpholinos with the compound. Fasudil treatment of crispant fish was also performed to examine the in vivo effect of blocking the predicted over-activity of the RhoA/ROCK pathway in the absence of Myo9aa/ab. This treatment significantly increased survival, indicating a positive action at 48 hpf and no overall detrimental development effects, despite the drug’s widespread action.

While crispants are a mosaic model, co-injection of 4 sgRNAs against the two genes targeted increases the chance of decreasing protein expression. Functional assessments of zebrafish revealed impaired movement at 24 and 48 hpf, similar to those reported for zebrafish lacking agrin [31]. Mice lacking agrin (Agrn^nmf380/nmf380^) also display poor motor control phenotypes, reflecting the importance of agrin in performing tasks relying on NMJ integrity [32]—and thus providing support for reduced agrin secretion, contributing to the pathomechanism of MYO9A-CMS. As impaired movements in zebrafish are not restricted to MYO9A or agrin knockdown and are present in a number of other zebrafish models of CMS, further evidence was obtained by the improvement of these behaviours by the application of NT1654. Of particular interest was the improvement in acceleration, which at this time point has been shown to be indicative of muscle contraction force [26]. A sarcopenia mouse model previously generated by increasing cleavage of agrin at the NMJ (by overexpression of neurotrypsin) displayed a reduced grip strength, which was restored with treatment using NT1654 [11].

Agrin secretion from the motor neuron during NMJ synaptogenesis is a critical step, without which AChR clusters do not form correctly [8]. In the *myo9aa/ab* crispant fish, there were fewer, larger AChR clusters, with an overall decrease in postsynaptic staining at 48 hpf. This corroborates observations of reduced myotomal AChR clustering in agrin-deficient zebrafish [31]. By 120 hpf, zebrafish lacking Myo9aa/ab exhibited an increase in number but decrease in average size of AChR clusters, as compared to wild-type fish. This also reflects observations in the *Agrn* KO mouse, which exhibits a number of small AChR clusters opposing nerve terminals during development in utero [8]. In support of the hypothesis that MYO9A-CMS is based on a reduction in agrin secretion, a number of the defects in NMJ morphology can be rescued by NT1654 treatment. For example, rescue of the presence of a choice point cluster of AChRs, the number of large clusters, and average AChR cluster area at 24 hpf was achieved. Application of NT1654 also increased the number of αBTx-positive clusters at 48 hpf, and myotome size at 120 hpf. Many of these improvements corroborate reported benefits of this compound in the aforementioned mouse model of sarcopenia [11]. The effect of NT1654 on neurons extending from the spinal cord or on number of terminals at later time points was unexpected, although application of agrin to in vitro cultures of PC12 cells and chick retinal neurons has been shown to mediate FGF2-induced neurite extension [33]. Furthermore, in the opposite scenario, removal of agrin from zebrafish has been shown to cause truncated primary motor axons during development, as well as erratic trajectories of these axons across the myotome, consistent with our findings [31]. Similar effects of abnormal axonal extensions are also observed in the diaphragm muscle of a murine model of agrin deficiency (AGZ) [8]. In the future, it would be interesting to identify whether the effects on motor neurons are limited to primary motor neurons or also involve secondary motor neurons in the zebrafish, which coordinate different forms of swimming. As primary motor neurons are associated with fast swimming responses, it is likely that this subtype is affected in our model; reflected in our movement data, older fish would be required to assess the secondary motor neurons which mediate slow, rhythmic swimming [24,25].

While the main hypothesis for the mode of action for MYO9A-loss is a reduction in agrin secretion from the nerve, previous analysis of MYO9A knockdown NSC-34 cells and the current study on zebrafish reveal effects on nerve and presynaptic nerve terminal morphology [5]. This could be related to cytoskeletal disruption due to RhoA over-activity, as application of Y-27632 was able to ameliorate some cytoskeletal defects in NSC-34 cells, and therefore improve intracellular trafficking [5]. Furthermore, treatment of zebrafish with the ROCK inhibitor, fasudil, was found to provide some improvements to the NMJ, including all disrupted features identified at 24 hpf—such as neurite extension—corroborating reports that ROCK inhibition can extend neurites in vitro [34]. The size of myotomes in the fish was also returned to wild-type size by fasudil treatment, correlating with results demonstrating a positive effect of fasudil application on muscle fibre size in an SMA mouse model [35]. These results highlight an important role for the RhoA/ROCK pathway in the phenotype of Myo9aa/ab-depletion in early zebrafish development, and it is possible that the improvements observed are due to fasudil in vivo act upstream of agrin release, as outlined in Figure 6. The lack of continued improvement to NMJ phenotype by fasudil throughout the time period assessed could be attributed to a number of causes. There may be a requirement for fine modulation of dosage for fasudil throughout development due to its widespread action and the likely differing contribution of the RhoA/ROCK pathway at different developmental stages. It could also be that effects of Myo9aa/ab at the NMJ are not mediated by the ROCK pathway as development proceeds, and instead are linked to other functions of this protein such as cross-linking actin filaments, or as yet unidentified interactions with other pathways of impact on the NMJ [36]. Nevertheless, the movement benefits due to fasudil treatment suggest there may be modest improvements in release/functionality at the NMJ that are not detectable by the morphological changes measured here.

Studying crispants has provided the benefit of assessing 120 hpf fish, which display innervation patterns similar to those observed in adult fish [27], which was not possible with MOs. Agrin has been shown to induce ectopic, fully differentiated postsynaptic compartments anywhere on the muscle, including insertion of AChRs [37]. While there is an increase in cluster number per 100 µm^2^ at 48 hpf in NT1654-treated crispants, this is only to the level observed in wild-types. However, at 120 hpf, there is a further increase in cluster number, which may represent ectopic AChRs. Colocalisation analysis is a useful metric, and as αBTx is very specific to postsynaptic AChRs, it is expected that the majority of the signal would be overlaid by SV2 after full innervation is complete (in the absence of ectopic clusters) and all prepatterned receptors are incorporated. Conversely, antibodies against SV2 can detect presynaptic vesicles that are localised along the nerve during development/transport, therefore may not necessarily all co-occur with post-synaptic αBTx [27]. Colocalisation analysis between AChRs and SV2 revealed no significant differences in co-occurrence between genotypes/treatment groups, indicating that around 70% of the AChR clusters identified were overlaid by nerves in all groups, which is similar to the proportions of colocalisation reported elsewhere and highlights the absence of ectopic cluster formation by NT1654 [38]. However, co-occurrence of SV2 with AChRs at 120 hpf is significantly reduced in crispant fish, as compared to wild-type, by around 30%. This could signify either an increase in presence of SV2 clusters or a decrease in AChRs. Analysis of NMJ morphology reveals a significant reduction in the average area of AChR clusters, thus supporting our hypothesis that Myo9aa/ab-depletion impairs secretion of agrin—and thus, clustering and stabilisation of AChRs. Treatment of 120 hpf fish with NT1654 significantly improved the impairment of postsynaptic colocalisation with the presynapse. An increase in the number of AChRs as compared to wild-type fish was also observed, but this was accompanied by a further decrease in the average area of clusters, thus indicating that the improvement in co-occurrence due to NT1654 may be a result of increased receptor insertion at novel sites rather than increased size of clusters present. NT1654 also significantly increased the average area of SV2-positive clusters which could contribute to the improved alignment of pre and postsynaptic NMJ components.

The evidence presented here supports a role for MYO9A at the NMJ, and the view that its absence or dysfunction is a cause of CMS. While many avenues for exploration of the precise function of MYO9A at the NMJ remain open, including a role in the postsynapse, an action on RhoA/ROCK and agrin secretion have been demonstrated. In the future, it will be important to confirm that the morphological defects identified in zebrafish manifest as impaired signal transmission at the NMJ and, thus, underpin a CMS phenotype. Furthermore, there is potential for the use of NT1654 in a clinical setting, including in MYO9A-CMS and other disorders in which agrin dysfunction is suspected.

## Figures and Tables

**Table 1:** NMJ morphology of *myo9aa/ab* crispant zebrafish at 24 hpf. A number of features of developing NMJs were characterised in wild-type (*n* = 7 fish), *myo9aa/ab* crispant (*n* = 6 fish), NT1654-treated (0.15ng, *n* = 7 fish) crispant, and fasudil-treated (10 µm, *n* = 10 fish) crispant fish, encompassing both pre- and postsynaptic regions. The mean value, standard deviation (SD), and *p*-value are shown for each measurement, with treated fish compared to untreated crispants and wild-type zebrafish. Average area of αBTx and size of myotomes = One-way ANOVA. Number of clusters >20 µm^2^, length of neuron outgrowths past the choice point, and proportion of somites with a choice point cluster = Kruskal-Wallis test.

	Wild-Type		*myo9aa/ab* Crispant			*myo9aa/ab* Crispant + NT1654				*myo9aa/ab* Crispant + Fasudil			
NMJ feature	Mean	SD	Mean	SD	P value (vs untreated WT)	Mean	SD	P value (vs untreated WT)	P value (vs untreated crispant)	Mean	SD	P value (vs untreated WT)	P value (vs untreated crispant)
Length of neuron outgrowth past choice point (µm)	19.91	10.53	8.55	8.70	**	24.15	9.46	ns	**	16.95	9.64	ns	ns
Average area of αBTx clusters (µm^2^)	2.62	0.41	1.72	2.25	ns	6.10	1.84	**	***	2.22	1.36	ns	ns
Total area of αBTx clusters per 100 µm^2^ (µm^2^)	14.13	2.84	18.1	25.74	ns	28.6	7.16	ns	ns	14.51	9.81	ns	ns
Number of αBTx clusters >20 µm^2^	5.68	3.01	3.2	4.19	*	9.5	3.08	ns	**	3.6	3.78	ns	ns
Proportion of somites with a choice point cluster	0.97	0.08	0.32	0.41	**	1	0	ns	**	0.90	0.20	ns	*
Size of myotomes (µm^2^)	2863	800.6	1724	971.2	**	1145	192.8	***	ns	2722	604.1	ns	*

* *p* ≤ 0.05, ** *p* ≤ 0.01, *** *p* ≤ 0.001, ns = not significant.

**Table 2:** NMJ morphology of *myo9aa/ab* crispant zebrafish at 48 hpf. A number of features of developing NMJs were characterised in wild-type (*n* = 10 fish), *myo9aa/ab* crispant (*n* = 11 fish), NT1654-treated (0.15 ng, *n* = 7 fish) crispant, and fasudil-treated (10 µm, *n* = 10 fish) crispant fish, encompassing both pre- and postsynaptic regions. The mean value, standard deviation (SD), and *p*-value are shown for each measurement, with treated fish compared to untreated crispants and wild-type zebrafish. Number of SV2 clusters >20 µm^2^, % myosepta overlaid by motor neuron, and size of myotomes = One-way ANOVA. Number of SV2/αBTx clusters and total area per 100 µm^2^, and average size of SV2/αBTx clusters = Kruskal-Wallis test.

	Wild-Type		*myo9aa/ab* Crispant			*myo9aa/ab* Crispant + NT1654				*myo9aa/ab* Crispant + Fasudil			
NMJ feature	Mean	SD	Mean	SD	P value (vs untreated WT)	Mean	SD	P value (vs untreated WT)	P value (vs untreated crispant)	Mean	SD	P value (vs untreated WT)	P value (vs untreated crispant)
No. of SV2 clusters per 100 µm^2^	3.72	0.85	2.53	1.74	**	4.48	1.96	ns	***	1.15	0.28	***	*
Avg area SV2 clusters (µm^2^)	2.17	1.25	3.44	2.53	**	1.77	0.89	ns	**	3.99	2.02	***	ns
Total area of SV2 clusters per 100 µm^2^ (µm^2^)	8.25	1.49	5.35	2.03	**	6.83	1.75	ns	ns	4.24	1.12	***	ns
Number of SV2 clusters >20 µm^2^	2.10	1.86	1.47	1.12	ns	1.79	1.53	ns	ns	1.72	1.14	ns	ns
% myosepta overlaid by motorneuron	79.0	15.47	64.90	22.15	ns	71.54	23.68	ns	ns	81.50	14.35	ns	ns
No. of αBTx clusters per 100 µm^2^	3.10	0.60	1.73	1.02	***	2.63	0.63	ns	*	1.18	0.29	***	ns
Avg area αBTx clusters (µm^2^)	1.64	0.46	3.12	2.27	***	1.33	0.36	ns	***	3.95	0.93	***	**
Total area of αBTx clusters per 100 µm^2^ (µm^2^)	8.00	1.96	4.38	2.1	**	3.43	0.81	**	ns	4.11	1.03	**	ns
Number of αBTx clusters >20 µm^2^	1.70	1.39	0.94	0.90	ns	0.64	0.93	*	ns	1.58	1.08	ns	ns
Size of myotomes (µm^2^)	5257	911	4574	1211	ns	4573	719	ns	ns	4646	1306	ns	ns

* *p* ≤ 0.05, ** *p* ≤ 0.01, *** *p* ≤ 0.001, ns = not significant.

**Table 3:** NMJ morphology of *myo9aa/ab* crispant zebrafish at 120 hpf. A number of features of developing NMJs were characterised in wild-type (*n* = 10 fish), *myo9aa/ab* crispant (*n* = 7 fish), NT1654-treated (0.15 ng, *n* = 8 fish) crispant, and fasudil-treated (10 µm, *n* = 10 fish) crispant fish, encompassing both pre- and postsynaptic regions. The mean value, standard deviation (SD), and p-value are shown for each measurement, with treated fish compared to untreated crispants and wild-type zebrafish. Number of SV2/αBTx clusters >20 µm^2^, % myosepta overlaid by motor neuron, total SV2/αBTx area per 100 µm^2^, number SV2 clusters per 100 µm^2^, average area of SV2 clusters, and size of myotomes = Kruskal-Wallis test.

	Wild-Type		*myo9aa/ab* Crispant			*myo9aa/ab* Crispant + NT1654				*myo9aa/ab* Crispant + Fasudil			
NMJ feature	Mean	SD	Mean	SD	P value (vs untreated WT)	Mean	SD	P value (vs untreated WT)	P value (vs untreated crispant)	Mean	SD	P value (vs untreated WT)	P value (vs untreated crispant)
No. of SV2 clusters per 100 µm^2^	3.44	0.63	5.14	1.55	ns	3.49	0.41	ns	ns	2.0	0.55	*	***
Avg area SV2 clusters (µm^2^)	1.02	0.18	0.96	0.21	ns	1.21	0.19	ns	*	4.27	1.46	***	***
Total area of SV2 clusters per 100 µm^2^ (µm^2^)	4.35	2.74	4.72	1.22	ns	4.21	0.78	ns	ns	7.93	2.63	**	ns
Number of SV2 clusters >20 µm^2^	0.73	0.73	0.14	0.38	ns	0.65	0.48	ns	ns	5.05	3.96	**	***
% myosepta overlaid by motorneuron	100	0	90.7	17.0	ns	100	0	ns	ns	100	0	ns	ns
No. of αBTx clusters per 100 µm^2^	4.39	0.94	5.68	1.28	*	7.09	3.04	*	ns	1.65	0.56	***	***
Avg area αBTx clusters (µm^2^)	1.18	0.13	0.82	0.23	***	0.48	0.12	***	**	2.04	0.69	***	***
Total area of αBTx clusters per 100 µm^2^ (µm^2^)	6.23	3.85	5.28	1.46	ns	5.11	1.55	ns	ns	3.41	1.57	*	ns
Number of αBTx clusters >20 µm^2^	1.22	1.30	0.15	0.24	ns	1.13	1.0	ns	ns	1.10	0.84	ns	*
Size of myotomes (µm^2^)	9977	1452	6256	1930	**	8785	1302	ns	ns	6838	2185	*	ns

* *p* ≤ 0.05, ** *p* ≤ 0.01, *** *p* ≤ 0.001, ns = not significant.

## Supplementary Materials

The following are available online at https://www.mdpi.com/2073-4409/8/8/848/s1, Figure S1: Genomic sequence of CRISPR-targeted myo9aa and myo9ab, Table S1: sgRNA sequences used for CRISPR/Cas9-mediated gene modification in zebrafish, Table S2: Annealing reaction for sgRNA oligo and universal bottom strand, Table S3: Thermocycler program for annealing of sgRNA and universal bottom strand, Table S4: Reaction mixture for zebrafish injection with sgRNA and Cas9 protein.
