# Supplementary material for: Modulation of Agrin and RhoA Pathways Ameliorates Movement Defects and Synapse Morphology in MYO9A-Depleted Zebrafish
Source: Cells. 2019 Aug 7;8(8):848. doi: 10.3390/cells8080848 (PMC6721702; doi:10.3390/cells8080848)
Supplement: Supplementary file 1 [file cells-08-00848-s001.pdf]

Gene	Target site sequence	Promoter + sgRNA + universal primer sequence	Off-targets	CRISPRscan efficacy score
myo9aa	AGGAGTTCGGTGGGGAGGAGTGG	taatacgactcactataGGGAGTTCGGTGGGGAGGAGgttttagagctagaa	0	79
myo9aa	GCGCTGGACGGGATAATCGGTGG	taatacgactcactataGGGCTGGACGGGATAATCGGgttttagagctagaa	0	77
myo9ab	GTGTTTCAGATCCTGTCCATTGG	taatacgactcactataGGGTTTCAGATCCTGTCCATgttttagagctagaa	0	69
myo9ab	AGTGCAGACTTGGAGCCCAGCGG	taatacgactcactataGGTGCAGACTTGGAGCCCAGgttttagagctagaa	0	78
Tyr	GGACTGGAGGACTTCTGGGG	taatacgactcactataGGACTGGAGGACTTCTGGGGgttttagagctagaa	0	69

Table 1. sgRNA sequences used for CRISPR/Cas9-mediated gene modification in zebrafish. Sequence of target site and target site including promoter and universal binding sequence are shown. Off targets refers to other areas of the genome the sgRNA may bind. The higher the CRISPRscan score (out of 100), the higher the predicted efficacy of that sgRNA.

Component	1x Volume (µl)
5x My Taq Buffer	5
gRNA target oligo (100µM)	2
Universal strand ultramer (100µM)	2
My Taq DNA polymerase	0.2
Nuclease free water	15.8
Total volume	25

Table 2. Annealing reaction for sgRNA oligo and universal bottom strand.

Temperature (°C)	Time (mins)
95	5
89	0.25
83	0.25
77	0.25
71	0.25
65	0.25
59	0.25
53	0.25
50	10
72	10
4	∞

Table 3. Thermocycler program for annealing of sgRNA and universal bottom strand.

Component	1x volume (µl)
10x Cas9 Buffer (0.2M HEPES, 1M NaCl, 50mM $MgCl_2$, 1mM EDTA)	1
EnGen Cas9 NLS Protein (20µM, NEB, MO646T)	3.58
RNA	3000ng - variable
2M KCL	1.5
0.5% Phenol red	1
RNase-free water	Up to 10
Total volume	10

Table 4. Reaction mixture for zebrafish injection with sgRNA and Cas9 protein.

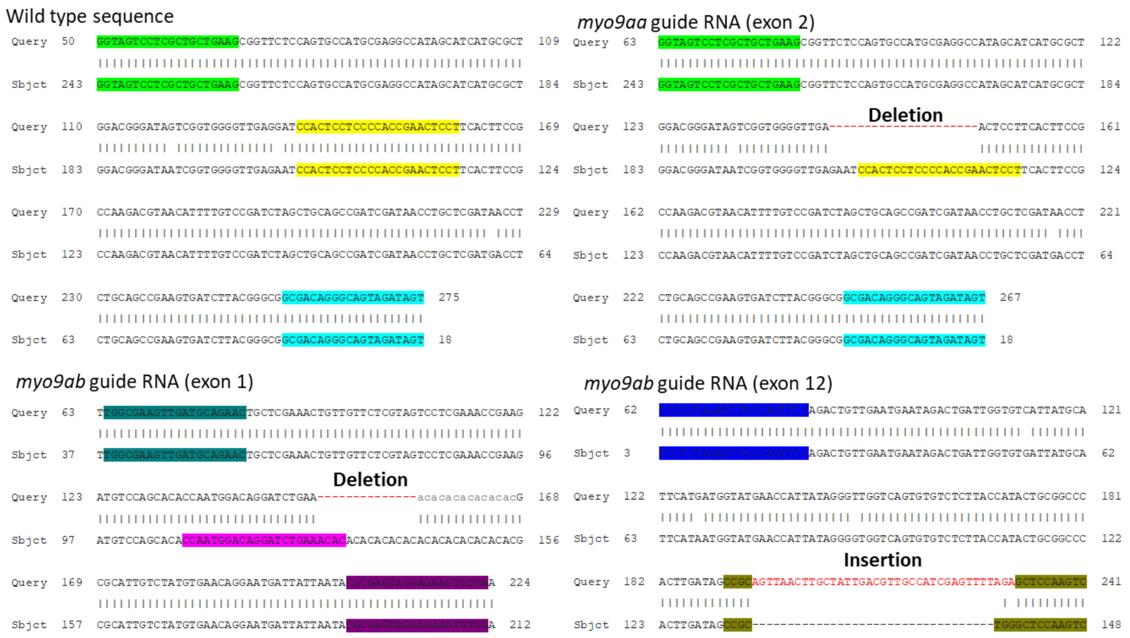

Figure 1. Genomic sequence of CRISPR-targeted myo9aa and myo9ab. The wild-type sequence for myo9aa is shown in the first panel, with the presence of a deletion in the target region of the sgRNA for exon 2 (highlighted yellow) shown in the top right. The bottom left panel shows a deletion induced by a sgRNA against exon 1 of myo9ab (pink), and in the bottom right panel an insertion can be observed (green) in exon 12 of myo9ab. The other highlighted regions show the location of the sequencing primers.
